# Editorial: Genetics, Genomics and -omics of Thermophiles, Volume II

**DOI:** 10.3389/fmicb.2022.879450

**Published:** 2022-03-31

**Authors:** Kian Mau Goh, Edgardo Rubén Donati, Rajesh Kumar Sani, Kok-Gan Chan

**Affiliations:** ^1^Department of Biosciences, Universiti Teknologi Malaysia, Johor Bahru, Malaysia; ^2^Facultad de Ciencias Exactas, Centro de Investigación y Desarrollo en Fermentaciones Industriales, CINDEFI (CCT La Plata-CONICET, UNLP), La Plata, Argentina; ^3^Department of Chemical and Biological Engineering, South Dakota School of Mines and Technology, Rapid City, SD, United States; ^4^Division of Genetics and Molecular Biology, Faculty of Science, Institute of Biological Sciences, University of Malaya, Kuala Lumpur, Malaysia; ^5^International Genome Centre, Jiangsu University, Zhenjiang, China

**Keywords:** CAZyme, comparative genomics, hot springs 16S rRNA, metagenomes, *Parageobacillus*, *Tepidiphilus*

There are many types of thermophilic prokaryotes, eukaryotes, and viruses that reside in heated environments such as hot springs and oil reservoirs. Comparative genomics and transcriptomics approaches, 16S rRNA marker sequencing (metataxonomy), shotgun metagenomes, and genomics help us gain a better understanding of thermophiles. An e-book with one review and ten research articles was published as Volume I of this Research Topic in 2017 (Goh et al., 2017). Volume II of this Research Topic includes four original articles.

To date, NCBI BioProject has around 2,000 submissions related to hot spring microbial taxonomics or metagenomics. To our knowledge, very few sites containing plant litter or plant biomass have been studied (Vishnivetskaya et al., 2015; Lee et al., 2018). A recent report examined microbial communities associated with four human-use Indian Deulajhari spring clusters (43–70°C, circumneutral pH) surrounded by Pandanus plants (Dixit et al.). The taxonomy of microbial phyla and the alpha-diversity were similar among the sites. Total organic carbon (TOC) in Tapta Kunda sites was significantly higher than Hima and Labakusha Kunda sites, likely because of leaf degradation. A canonical correlation analysis revealed that *Thermoanaerobacter, Desulfovibrio*, Candidatus *Solibacter*, and *Dehalogenimona* were positively correlated with TOC levels. Authors predicted the carbohydrate-degrading ability of these thermophilic bacteria using Tax4fun and PICRUSt. Hopefully, shotgun metagenomic data of Deulajhari hot springs will be made available soon as microbiota and carbohydrate-acting enzymes in geothermal springs rich in plant biomass are more complex than those without lignocellulose feed (Reichart et al., 2021; Liew et al., 2022).

Several factors can influence the microbial community of a hot spring, such as its temperature and pH. The second article investigated As Burgas (AS) and Muiño da Veiga (MDV) hot springs in Spain using short-read metagenome sequencing. A comparison of the microorganisms and community structure of nearby springs was conducted in order to measure the influence of environmental conditions on microbial diversity (DeCastro et al.). AS and MDV are 5 km away within Galicia. Bacteria were dominated (94–97%) in these geothermal springs while archaea accounted only <5%. The article also discovered DNA sequences from eukaryotes and viruses. Proteobacteria (~70%), Deinococcus-Thermus and Firmicutes were the dominant groups in AS microbiome. This result is in agreement with other studies that found that the Proteobacteria dominate moderate- to high-temperature geothermal springs. Temperature, pH, and Na ion values were similar at both sites; however, other abiotic parameters differed. For instance, probably due to higher sulfate in the water, MDV hot spring was more abundant in Aquificae (*Sulfurihydrogenibium* spp.) and Nitrospirae (*Thermodesulfovibrio* spp.). Aquificae is a more common phylum in the MDV thermal springs. The Aquificae are often found in other high temperature springs as well. We hope that the current microbial diversity study, as well as other phylogenetic data, can be compiled so that we can get a better understanding on how microbiome diversity scales based on the diversity-area relationship models, random forest models, or neural network models.

During the flooding process of petroleum reservoirs, water is heated in the ground. In a recent publication on this Research Topic, 93°C was measured for the recovered water from an oil field in the Orinoquia region of Colombia, South America (Bedoya et al.). Before reusing water to flood reservoirs, the industry introduces biocides glutaraldehyde and tetrakis-hydroxymethyl-phosphonium sulfate to reduce the microbial load. When bacteria are not dealt with properly during the process, they can cause clogs and corrosion of pipes along with the production of harmful gases such as hydrogen sulfide. Comparing microbial metagenome data from injected and produced water samples was undertaken by the authors (Bedoya et al.). Only a few microbial operating taxonomy units (OTUs) could survive the harsh environment (high temperatures, high pressures, biocides, anoxia, high pressure, heavy metals, etc.). A high-quality *Tepidiphilus* sp. UdeAICP_D1 draft genome was generated using the metagenome-assembled genome approach. It has been documented that oil reservoirs contain low levels of *Tepidiphilus*, but the two samples studied in this study contained the same OTU that dominated (>75%) bacterial populations. Analyzing the genome of UdeAICP_D1 in relation to other *Tepidiphilus* spp. may indicate that UdeAICP_D1 has high-performance stress and toxic response mechanisms. UdeAICP_D1 contains a number of genes related to cell division, resistance, and nodulation, type II toxin-antitoxin systems, transporters, biofilm formation, and other potential strategies. Because *Tepidiphilus* has not been properly studied, it is vital to study its resistance to biocides. Furthermore, studying its genome might allow the industry to discover alternative biocides that could be used against this species.

Microbiological gasification is a promising alternative energy source. During the water-gas shift (WGS) reaction, carbon monoxide (CO) is transformed into carbon dioxide (CO_2_) and hydrogen (H_2_) by the reaction with water (H_2_O). The enzymes, carbon monoxide dehydrogenase and hydrogenase, are involved in biological WGS gasification reactions (Alfano and Cavazza, 2018). In a modified Luria-Bertani medium supplemented with minerals, Aliyu and her team grew several strains of *Parageobacillus thermoglucosidasius* separately under an initial gas combination of 1:1 CO and air for 70 h at 60°C. *P. thermoglucosidasius* strain Kp1013 was the only one that produced biohydrogen under the conditions (Aliyu et al.); however, other examined strains could produce biohydrogen in another experimental setup (Mohr et al., 2018). In the genome-wide analysis of *Parageobacillus*, nickel and iron-binding and transport proteins were found to be essential to Ni-Fe hydrogenase activity; therefore, these proteins may be associated with biohydrogen production by the bacteria. A more comprehensive study is needed to determine whether *Parageobacillus* is an appropriate thermophilic candidate for generating biohydrogen at industrial scale.

Research on thermal environments and thermophiles continues to provide new insights into microorganisms in extreme environments. This e-book is intended to encourage members of the research community to use bioinformatics and -omics, in combination with enrichment cultivation approaches to better understand the biology of thermal environments and thermophiles.

## Author Contributions

All authors listed have made a substantial, direct, and intellectual contribution to the work and approved it for publication.

## Funding

ERD would like to acknowledge the financial support provided by the ANPCyT (PICT-2018-02664), Argentina. K-GC. thanks the financial support from the FRGS grant (FP022-2018A). KMG is grateful for funding received from Malaysia FRGS grants 5F241 and 5F245 with the respective reference code FRGS/1/2019/STG03/UTM/02/1 and FRGS/1/2019/STG04/UTM/02/4.

## Conflict of Interest

The authors declare that the research was conducted in the absence of any commercial or financial relationships that could be construed as a potential conflict of interest.

## Publisher's Note

All claims expressed in this article are solely those of the authors and do not necessarily represent those of their affiliated organizations, or those of the publisher, the editors and the reviewers. Any product that may be evaluated in this article, or claim that may be made by its manufacturer, is not guaranteed or endorsed by the publisher.
